# Image-based methods for phase estimation, gating and temporal super-resolution of cardiac ultrasound

**DOI:** 10.1109/TBME.2018.2823279

**Published:** 2018-04-24

**Authors:** Deepak Roy Chittajallu, Matthew McCormick, Samuel Gerber, Tomasz J. Czernuszewicz, Ryan Gessner, Monte S. Willis, Marc Niethammer, Roland Kwitt, Stephen R. Aylward

**Affiliations:** Kitware Inc.; Kitware Inc.; Kitware Inc.; SonoVol; SonoVol; Dept. of Pathology and Laboratory Medicine at University of North Carolina - Chapel Hill; Dept. of Computer Science and the Biomedical Research Imaging Center (BRIC) at University of North Carolina – Chapel Hill; Dept. of Computer Science at University of Salzburg; Kitware Inc.

**Keywords:** Ultrasound, Echocardiography, Cardiac, Phase estimation, Gating, Temporal Super-resolution

## Abstract

**Objective:**

Ultrasound is an effective tool for rapid non-invasive assessment of cardiac structure and function. Determining the cardiorespiratory phases of each frame in the ultrasound video and capturing the cardiac function at a much higher temporal resolution is essential in many applications. Fulfilling these requirements is particularly challenging in preclinical studies involving small animals with high cardiorespiratory rates, requiring cumbersome and expensive specialized hardware.

**Methods:**

We present a novel method for the retrospective estimation of cardiorespiratory phases directly from the ultrasound videos. It transforms the videos into a univariate time-series preserving the evidence of periodic cardiorespiratory motion, decouples the signatures of cardiorespiratory motion with a trend extraction technique, and estimates the cardiorespiratory phases using a Hilbert transform approach. We also present a robust nonparametric regression technique for respiratory gating and a novel kernel-regression model for reconstructing images at any cardiac phase facilitating temporal super-resolution.

**Results:**

We validated our methods using 2D echocardiography videos and electrocardiogram (ECG) recordings of 6 mice. Our cardiac phase estimation method provides accurate phase estimates with a mean-phase-error-range of 3–6% against ECG derived phase and outperforms three previously published methods in locating ECGs R-wave peak frames with a mean-frame-error-range of 0.73–1.36. Our kernel-regression model accurately reconstructs images at any cardiac phase with a mean-normalized-correlation-range of 0.81–0.85 over 50 leave-one-out-cross-validation rounds.

**Conclusion and Significance:**

Our methods can enable tracking of cardiorespiratory phases without additional hardware and reconstruction of respiration-free single cardiac-cycle videos at a much higher temporal resolution.

## I. Introduction

Cardiovascular disease is the leading cause of death worldwide and ultrasound is an effective tool for rapid noninvasive assessment of cardiac structure and function [1]–[3]. Knowledge of the phase or location of each video frame within the cardiac and/or respiratory cycle is essential in many applications (e.g. gating [4], quiescence detection [5], 3D reconstruction [6]) and the ability to capture cardiac function at a high temporal resolution is vital for accurate diagnosis (e.g. wall and valve motion assessment) [3]. Typically, cardiac phase is tracked by a simultaneously acquired ECG or pulseoximetry signal and respiratory phase is tracked by motion of markers placed on the subject’s body [6]–[8]. Setting up such hardware is cumbersome particularly in pre-clinical studies involving small animals [1]. Moreover, frame rates of affordable commercial ultrasound transducers fall short in imaging small animals such as mice with high heart (310–840 BPM) and respiration (80–230 BPM) rates [9], [10]. In particular, there are very short-lived events within the cardiac cycle (specifically peak systole and diastole) that require ultrahigh temporal sampling. Previous studies have shown that frame rates of up to 2000–2700 Hz (250–350 frames/cardiac cycle) were required to capture the minute deformations for cardiac strain estimation [11]. To achieve such high frame rates, specialized hardware (e.g. retrospective ECG gating, plane wave imaging) has typically been utilized [3], [9], [10].

### Contributions.

In this paper, we present a method for retrospective estimation of instantaneous cardiac and respiratory phases directly from cardiac ultrasound videos. We also present a robust non-parametric regression technique for gating out respiratory frames and a kernel regression model for reconstructing images at any cardiac phase to facilitate temporal super-resolution.

### Related prior work.

Previous work on the estimation of cardiac and/or respiratory phases directly from ultrasound echocardiography videos is limited. In [14], Karadayi *et al*. compute a signal of the x- or y-coordinate of the center-of-mass of each frame, use a band-pass filter to remove frequencies outside the cardiac range, determine the dominant frequency in the periodogram, and apply matched filtering using single-period sine and cosine signals at the dominant frequency to estimate the instantaneous cardiac phase. However, the center-of-mass signals may not be reliable in all scenarios. In [4], Sundar *et al*. compute a signal of phase correlation between consecutive frames and apply a band-pass and low-pass filter to obtain an estimate of the instantaneous cardiac and respiratory phases, respectively. Phase correlation encodes global translation in the image plane and cannot model out-of-plane motion of the beating heart present in our data. In [15], Wachinger *et al*. use a manifold learning or non-linear dimensionality reduction technique called Laplacian Eigenmap to learn the low-dimensional manifold of the image sequence embedded in high-dimensional space and project the images onto the first eigen direction of the Laplacian of the image similarity graph to obtain a 1D signal encoding respiratory motion. In [16], Panayiotou *et al*. use a series of image filtering operations to obtain a binary mask of pixels predominantly affected by cardiac/respiratory motion, apply a linear dimensionality reduction technique called principal component analysis (PCA) on the intensities of image pixels within the binary mask, project the images onto the principal directions with high variation to extract 1D signals encoding cardiac/respiratory motion, and postprocess these signals by suppressing undesired frequencies in the frequency domain. While the aforementioned approaches based on manifold learning and masked PCA are promising and generally applicable, in this paper, we present a method that can estimate the cardiac phases more accurately. Instead of using dimensionality reduction methods such as PCA or manifold learning to implicitly derive intermediate representations decoupling the cardiac and respiratory motion, our method explicitly addresses this problem through a trend extraction technique that works directly on the inter-frame similarity in the original high-dimensional space. It then goes a step further and estimates the instantaneous cardiac and respiratory phases of each frame using a Hilbert transform approach that is shown to be less sensitive to small short-lived fluctuations [13].

## II. Method

**Fig. 1.**
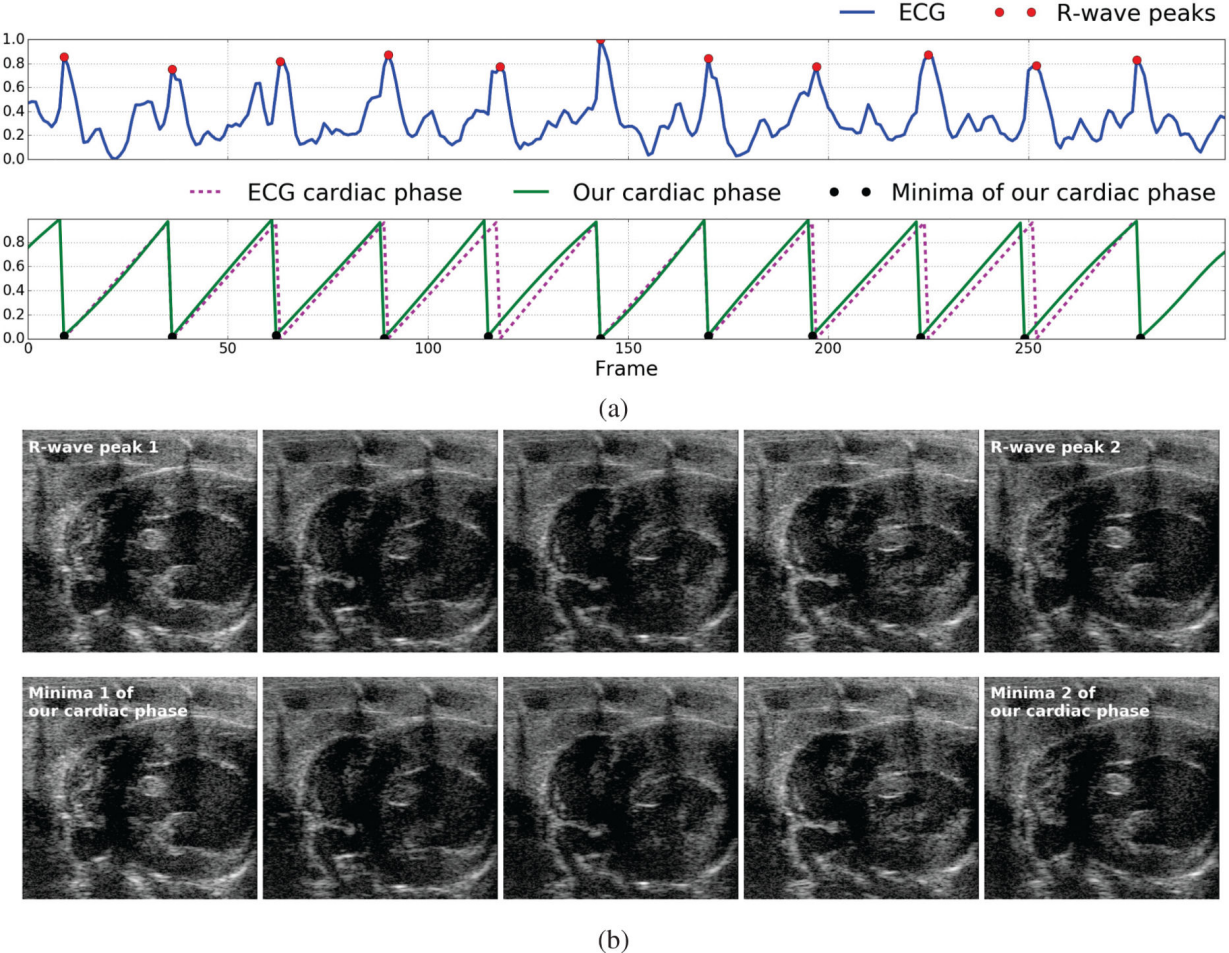
Illustration of the close match between the cardiac phase derived from the ECG signal and the cardiac phase estimated using our method: (a) ECG signal simultaneously acquired with the image sequence (blue) overlaid with peaks of the R-wave in each cardiac cycle (red dot/circle), cardiac phase derived from the ECG signal (Pink dashed line) through linear interpolation between R-wave peaks [12], [13], and the cardiac phase estimated directly from the image data using our method (green solid line) overlaid with the corresponding minima (black dot/circle), (b) Five video frames evenly spaced in time between the first and second R-wave peaks of the ECG signal (top-row) and the corresponding minima of the cardiac phase estimated using our method (bottom-row) constituting one cardiac cycle. Notice that the images in each column look very similar.

In this section, we present the theory underlying the proposed methods along with visual illustrations of the intermediate results to help understand the underlying concepts. In Section II-A, we describe our method for estimation of instantaneous cardiac and respiratory phases (Figure 1). In Section II-B, we present a robust method to exclude video frames with significant respiratory motion. In Section II-C, we present a kernel regression model for reconstructing images at any cardiac phase facilitating temporal super-resolution.

### A. Estimation of cardiac and respiratory phases

While there have been numerous efforts for the estimation of instantaneous phase and/or frequency in periodic univariate time series data [12], [13], [17]–[19], the methods that tackle this problem in a multivariate setting such as the case of cardiac ultrasound videos wherein thousands of variables (pixel intensities) are involved is limited. Our strategy is to transform this complex multivariate problem into a univariate one and take advantage of existing methods to solve the problem.

**Fig. 2.**
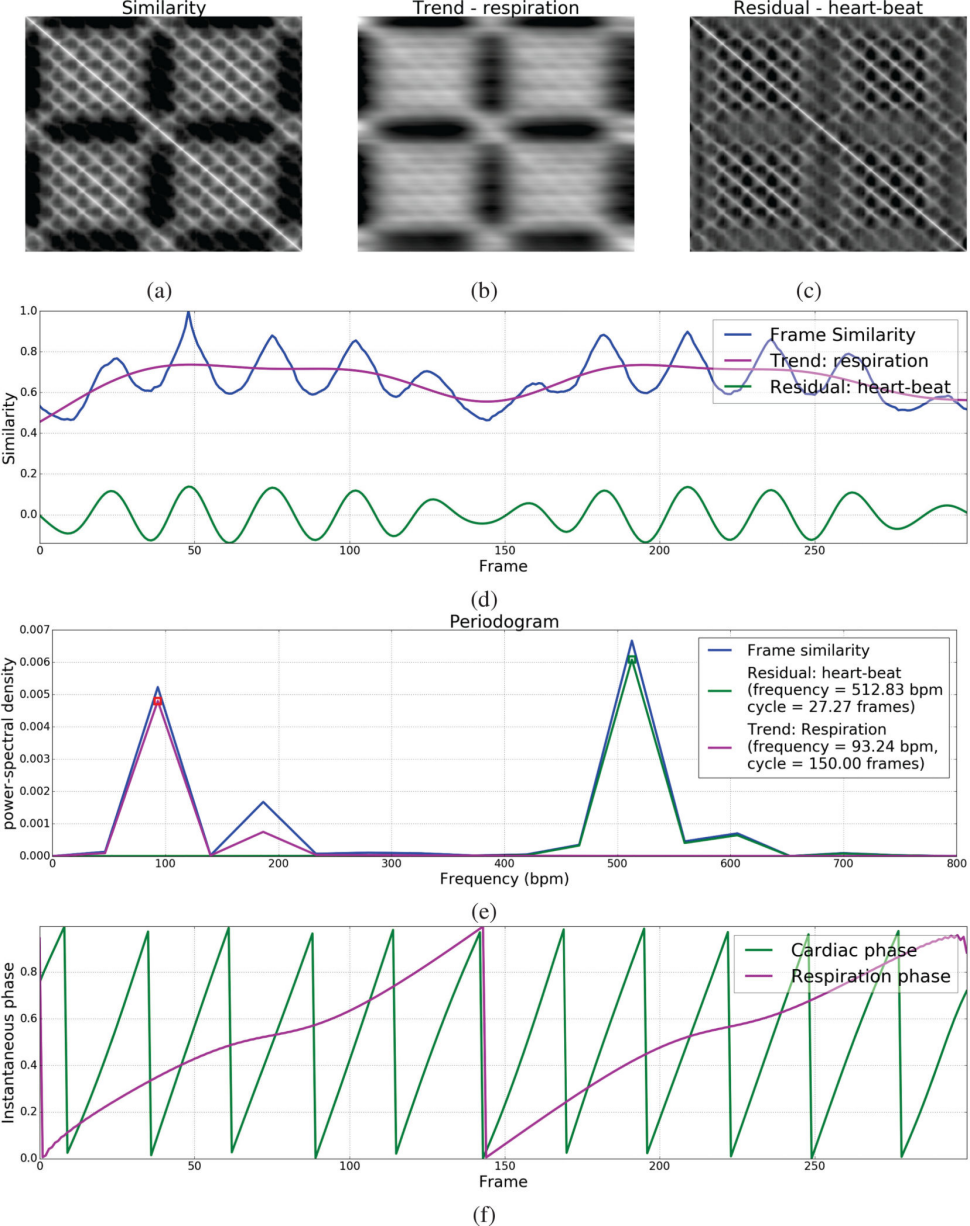
Illustration of our phase estimation method: (a) Inter-frame similarity matrix, (b) Trend matrix corresponding to respiratory motion, (c) Residual matrix corresponding to beating heart motion, (d) Frame similarity $\hat{u}(t)$ selected for phase estimation with associated trend/respiration ${\hat{\tau }}_{resp}$ and residual/heart-beat ${\hat{r}}_{heart}$ signals, (e) Periodogram of the frame similarity, residual/heart-beat and trend/respiration signals along with periodicity characteristics (e.g. frequency, cycle duration) calculated based on the dominant frequency, and (f) Instantaneous cardiac and respiratory phases estimated using the Hilbert transform.

We first compute the similarity between all pairs of images/frames in the given quasi-periodic image sequence containing *N* frames to create a symmetric matrix *S* ∈ *R*^*N*×*N*^ wherein the element *S*(*i,j*) is equal to the similarity between the *i*^*th*^ and *j*^*th*^ frame. Each row in the inter-frame similarity matrix *S* can now be seen as a univariate time series. The inter-frame similarity metric must be chosen such that this time series preserves the periodicity characteristics of cardiorespiratory motion in the original image sequence. Here, we use normalized correlation to quantify inter-frame similarity; but in principle, other image similarity metrics can be used [20]. Figure 2(a) shows the inter-frame similarity matrix of one of our cardiac ultrasound videos wherein the periodicity characteristics of low-frequency respiratory motion and high-frequency beating heart motion can be observed. Notice that the rows of this matrix appear to be a superposition of two near-sinusoidal signals with the periodic signatures of cardiac and respiratory motion, respectively.

Next, we use a trend extraction technique called the Hodrick-Prescott (HP) filter [21] to decouple the periodic signatures of cardiac and respiratory motions from the frame similarity signal *u*^*i*^(*t*) corresponding to each row *i* of the matrix *S* by decomposing it into a sum of two components: (i) lower frequency trend component $\tau_{resp}^{i}(t)$ with periodicity characteristic of only respiratory motion, and (ii) higher frequency residual component $r_{heart}^{i}(t)$ with periodicity characteristic of only beating heart motion. The HP filter performs the decomposition of $u^{i}(t)=\tau_{resp}^{i}(t)+r_{heart}^{i}(t)$ by solving the following optimization problem:

(1) $$\underset{\tau_{resp}^{i}(t)}{\arg\;\min}\;\sum_{t=1}^{N}{\left(u^{i}(t)-\tau_{resp}^{i}(t)\right)}^{2}+\lambda \sum_{t=1}^{N-1}{\left(\nabla^{2}\tau_{resp}^{i}(t)\right)}^{2}$$

where $\nabla^{2}\tau_{resp}^{i}(t)=\tau_{resp}^{i}(t+1)-2\tau_{resp}^{i}(t)+\tau_{resp}^{i}(t-1)$ is the second-order difference or derivative of the trend signal and *λ* is a penalty parameter to control the smoothness of the trend component. *λ* should be set to a value that results in a good separation between the periodograms or power-frequency distributions of the resulting trend and residual component signals. This may depend on the frame rate of the ultrasound video and the heart/respiration rate of the subject. We set *λ* = 6400 for all our experiments unless stated otherwise. Let *S*_*resp*_ and *S*_*heart*_ be the matrices whose rows contain the trend/respiratory and residual/heart-beat components (Figures 2(b,c)), respectively, of the frame similarity signal in the corresponding rows of matrix *S*. We then select the trend/respiratory and residual/heart-beat component signals corresponding to one of the rows in *S* for phase estimation. A natural question that arises now is which of these rows is a better choice for phase estimation. We devised a procedure to make this choice objective based on the notion that if the HP filter was successful in decoupling the periodic signatures of cardiac and respiratory motion, then the resulting trend/respiratory and residual/heart-beat component signals must be as near-sinusoidal or narrow-banded as possible. For each row *i* in the inter-frame similarity matrix *S*, we compute the periodogram or power-frequency distribution *p*_*i*_(*f*) of its heart-beat component signal, normalize it to sum to 1 to be treated as a probability distribution, and compute its entropy $E_{i}\left(p_{i}\right)=-\sum_{f}p_{i}(f)log\left(p_{i}(f)\right)$. The smaller this entropy value, the more narrow banded it would be. Hence, we select the row with the smallest entropy value. Let $\hat{u}(t)$ be the frame similarity signal of the selected row and let ${\hat{\tau }}_{resp}(t)$ and ${\hat{r}}_{heart}(t)$ be its trend and residual components (Figure 2(d)) that we will henceforth refer to as respiration and heart-beat signals, respectively. To suppress undesired frequencies, we apply a low-pass filter with a typical mice respiration cutoff frequency of 230 BPM to the respiration signal ${\hat{\tau }}_{resp}(t)$ and a band-pass filter within the typical mice heart frequency range of 310–840 BPM to the heart-beat signal ${\hat{r}}_{heart}(t)$.

Next, considering the narrow-band near-sinusoidal nature of the respiration and heart-beat signals (Figure 2(e)), we use the concept of the analytic signal involving the Hilbert transform as introduced by Gabor to estimate the instantaneous phase of each frame [12], [13], [22], [23]. Specifically, we compute the instantaneous phase ϕ(*t*) ∈ [−*π,π*) of a periodic time series *x*(*t*) to be the instantaneous phase of its analytic signal *A*_*x*_(*t*) = *x*(*t*)+*iH*_*x*_(*t*) involving its Hilbert transform *H*_*x*_(*t*) as follows: $\phi (t)=arctan\left(\frac{H_{x}(t)}{x(t)}\right)$ and map ϕ(*t*) from [−*π,π*) to [0,1). Let ${\hat{\phi }}_{heart}(t)$ and ${\hat{\phi }}_{resp}(t)$ denote the instantaneous cardiac and respiratory phases (Figure 2(f)) computed from the respiration and heart-beat signals, respectively.

### B. Gating out respiratory frames

Once the cardiac and respiratory phases of each frame have been estimated, they can be used to select or filter out frames from a desired part/point in the periodic cycle, a process commonly referred to as gating. In this section, we present a robust two-step method that uses these phase estimates to filter out video frames with significant respiratory motion.

**Fig. 3.**
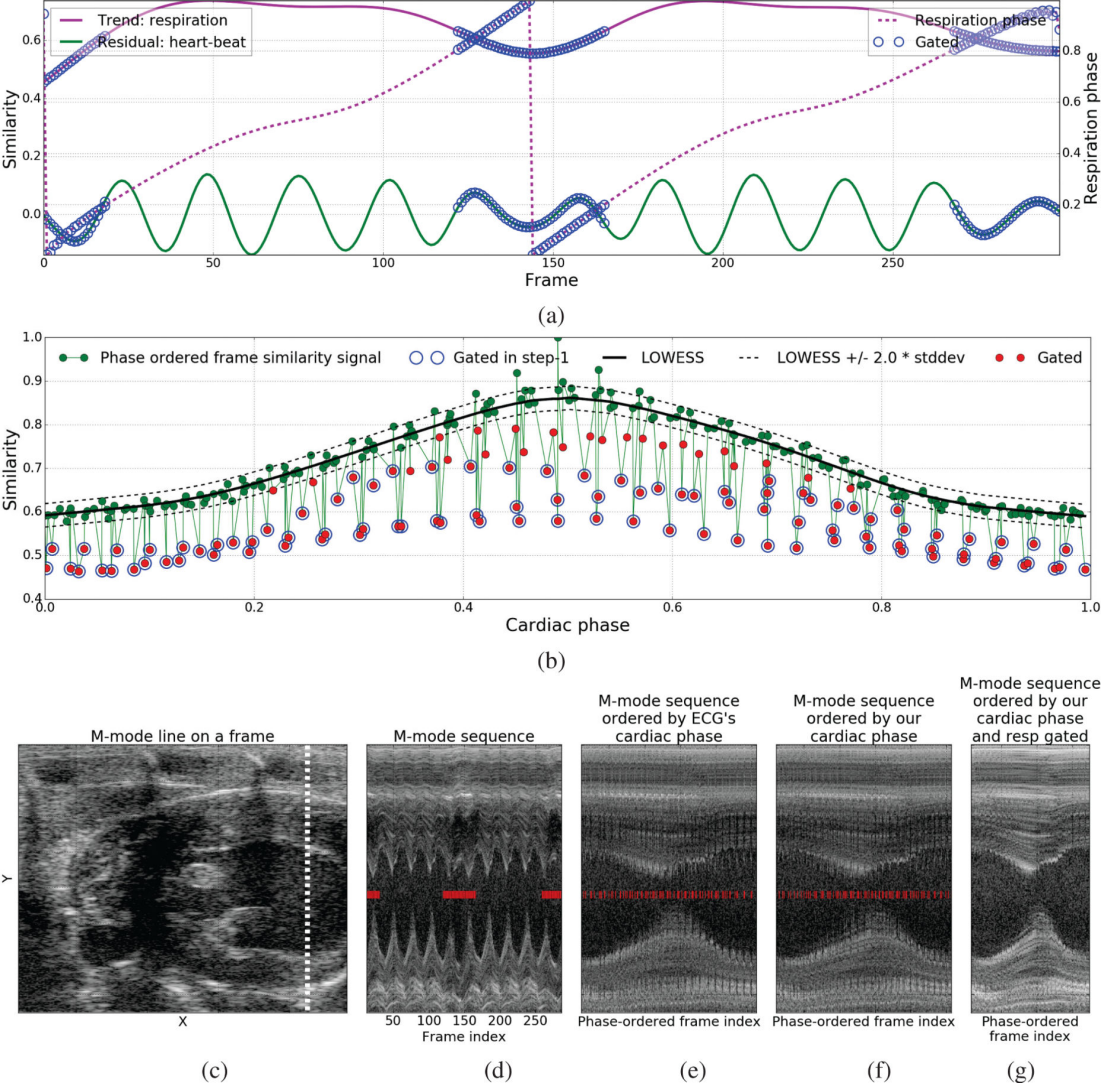
Illustration of the respiratory gating method: (a) The set of frames *F*_*cutoff*_ discarded (blue circles) in step-1 overlaid with the respiration ${\hat{\tau }}_{resp}(t)$, heart-beat ${\hat{r}}_{heart}(t)$, and respiratory phase ${\hat{\phi }}_{resp}(t)$ signals, (b) The frame similarity signal $\hat{u}(t)$ vs cardiac phase ${\hat{\phi }}_{heart}(t)$ overlaid with frames *F*_*cutoff*_ discarded in step-1 (blue circles), LOWESS fit *L*(*ϕ*_*heart*_) (black solid lines), upper and lower bounds or 95% confidence interval (*L*(*ϕ*_*heart*_)±2.0 ∗ ${\hat{\sigma }}_{L}$) of non-respiratory frames around the LOWESS fit (black dotted line), and frames *F*_*resp*_ (red circles) gated after step-2, (c) A frames from one of our cardiac ultrasound videos overlaid with the M-mode line shown in the next four images to the right, (d) M-mode frames in the order they appear in the input video, (e) M-mode frames ordered by cardiac phase derived from the ECG signal, (f,g) M-mode frames ordered by cardiac phase estimated using our method before and after respiratory gating. Overlaid red bar markers in (d-f) indicate the frames with heavy respiratory motion discarded by our respiratory gating technique.

In the first step, based on the observation that heaviest amount of the respiratory motion occurs within a short interval around the minima of the respiration signal ${\hat{\tau }}_{resp}(t)$ (pink curve in Fig. 2(d)) with respiratory phase ${\hat{\phi }}_{resp}(t)=0$, we perform a rough initial gating by discarding the frames $F_{cutoff}=\left\{t|{\hat{\phi }}_{resp}(t)<c\vee {\hat{\phi }}_{resp}(t)>(1-c)\right\}$ whose phase distance from ${\hat{\phi }}_{resp}(t)=0$ is below a specified cutoff value *c* = 0.2 chosen empirically. Figure 3(a) shows the discarded frames *F*_*cutoff*_ overlaid on the respiration ${\hat{\tau }}_{resp}(t)$, heart-beat ${\hat{r}}_{heart}(t)$, and respiratory phase ${\hat{\phi }}_{resp}(t)$ signals.

In the second step, we learn a regression function *L*(ϕ_*heart*_) : [0,1) → *R* to predict the frame similarity signal value for any cardiac phase by fitting a robust non-parametric regression model called Locally Weighted Regression (LOWESS) [24] to the dataset $\left\{\left({\hat{\phi }}_{heart}(t),\hat{u}(t)\right)|\forall t\notin F_{cutoff}\right\}$ containing the pair of the cardiac phase ${\hat{\phi }}_{heart}(t)$ and frame similarity signal $\hat{u}(t)$ values of all frames that do not belong to the set of frames *F*_*cutoff*_ discarded in the first step above. Given any cardiac phase, LOWESS regression takes k-nearest training samples based on their cardiac phase values and uses iterative weighted linear regression to predict the corresponding frame similarity signal value. This local fitting approach enables LOWESS regression to model a much wider class of functions than is possible with parametric approaches such as polynomial regression. Next, we compute a robust estimate of the standard deviation ${\hat{\sigma }}_{L}$ of non-respiratory frames around the LOWESS fit based on the median absolute deviation between the LOWESS fit $L\left({\hat{\phi }}_{heart}(t)\right)$ and frame similarity signal $\hat{u}(t)$ for all frames. Lastly, we gate out all frames $F_{resp}=\left\{t:\left|\hat{u}(t)-L\left({\hat{\phi }}_{heart}(t)\right)\right|>k\times {\hat{\sigma }}_{L}\right\}$ whose frame similarity signal value is below the LOWESS fit by more than *k* = 2.0 (95% confidence interval) times the standard deviation ${\hat{\sigma }}_{L}$ (Figure 3(b)). Figure 3(d) shows an m-mode view of one of our videos along the m-mode line shown in Figure 3(c). Figures 3(e,f) show the m-mode sequence ordered by the cardiac phase derived from the ECG signal and the cardiac phase estimated using our method, respectively, that look very similar. Notice the jaggedness/discontinuities along the horizontal direction at chamber wall in both these images that is caused by large movements induced by respiratory motion. Figure 3(g) shows the result obtained by ordering the m-mode sequence by the cardiac phase estimated using our method and gating out the respiratory frames using the method described above. Notice the significant reduction in the jaggedness/discontinuities after gating out or discarding the frames with heavy respiratory motion.

### C. Model to reconstruct images by cardiac phase

**Fig. 4.**
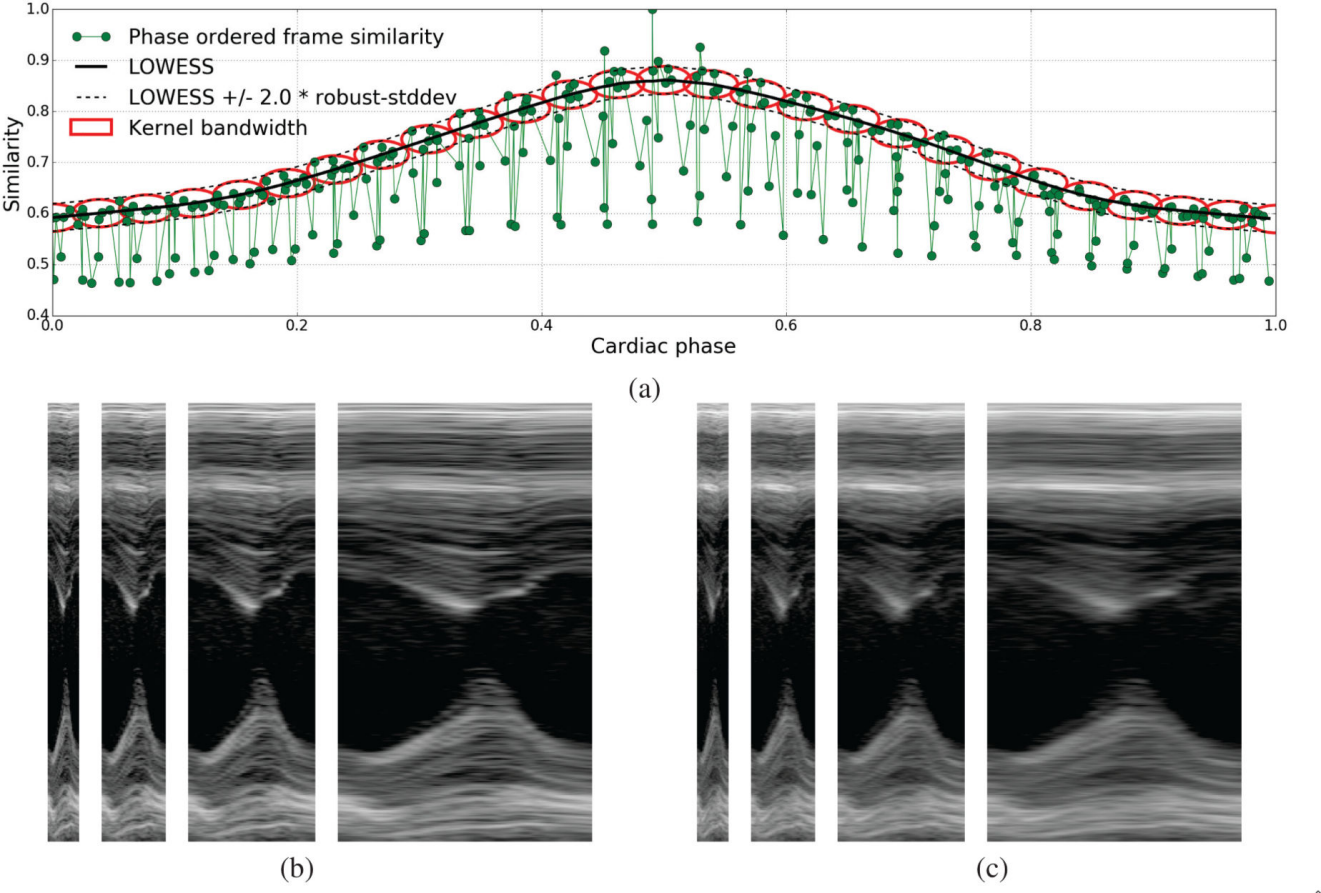
Illustration of temporal super-resolution using our kernel regression model: (a) Shows the frame similarity signal $\hat{u}(t)$ vs cardiac phase ${\hat{\phi }}_{heart}(t)$ overlaid with LOWESS fit *L*(ϕ_*heart*_) (black solid lines), upper and lower bounds or 95% confidence interval (*L*(ϕ_*heart*_)±2.0∗ *σ*_*L*_) of non-respiratory frames (black dotted lines), and the kernel bandwidth shown at a series of cardiac phases as red ellipses whose length along the phase and similarity axis is set to *σ*_ϕ_ and *σ*_*L*_ as described in Section II-C, (b,c) M-mode views of single cardiac cycle videos reconstructed at 1x (27 frames/cycle), 2x (54 frames/cycle), 4x (110 frames/cycle), and 8x (220 frames/cycle) temporal magnification using the NW kernel regression model with (left) the proposed bivariate kernel defined in both cardiac phase and frame similarity space vs (right) a univariate kernel defined in cardiac phase space only.

In this section, we present a kernel regression model to reconstruct the image at any cardiac phase. This model can then be used to generate a single-cycle video representative of the subject’s heart-beat at a higher temporal resolution from a low-frame rate video of multiple heart beats. Given a cardiac ultrasound video *I*(*t*) : {1*,...,N*} → *R*^*m*^ of *N* frames with *m* pixels each, we estimate the instantaneous cardiac phase ${\hat{\phi }}_{heart}(t)$ of each frame and compute the robust LOWESS fit *L*(ϕ_*heart*_) : [0,1) → *R* that maps cardiac phase to its frame similarity signal value as described in Sections II-A and II-B. We then use Nadarya-Watson (NW) kernel regression [25] to learn a function *M*(ϕ_*heart*_) : [0,1) → *R*^*m*^ that reconstructs the image for any cardiac phase ϕ using a kernel-weighted local average as follows:

(2) $$M\left(\phi_{heart}\right)=\frac{\sum_{t=1}^{N}K\left(\phi_{heart},{\hat{\phi }}_{heart}(t)\right)I(t)}{\sum_{t=1}^{N}K\left(\phi_{heart},{\hat{\phi }}_{heart}(t)\right)}$$

wherein we define the kernel $K\left(\phi_{heart},{\hat{\phi }}_{heart}(t)\right)=exp\left\{-\frac{{\left|\phi_{heart}-{\hat{\phi }}_{heart}(t)\right|}^{2}}{2\sigma_{\phi }^{2}}\right\}\times exp\left\{-\frac{{\left|L\left(\phi_{heart}\right)-\hat{u}(t)\right|}^{2}}{2\sigma_{L}^{2}}\right\}$ as the product of two radial-basis function (RBF) kernels. The first RBF kernel gives higher weights to images whose cardiac phase is closer (accounting for periodicity) to the target phase. Its bandwidth *σ*_ϕ_ is set equal to a constant *k*_ϕ_ = 0.4 times the median difference in cardiac phase between consecutive frames of the given image sequence. The second RBF kernel gives higher weights to images whose frame similarity signal value is close to the LOWESS prediction *L*(ϕ_*heart*_). Its bandwidth *σ*_*L*_ is set equal to a constant *k*_*L*_ = 2.0 times the robust estimate of standard deviation ${\hat{\sigma }}_{L}$ of non-respiratory frames (∀*t /*∉ *F*_*resp*_) around the LOWESS fit. As a result of this, frames with heavy respiratory motion will get very low weight values even if their cardiac phase is similar to the target phase. Figure 4(a) shows bandwidth of the kernel in phase and similarity space. The model *M*(ϕ_*heart*_) can now be used to reconstruct a single-cycle video representative of the subject’s heart-beat by generating images at any desired resolution/sampling of phases in the range [0,1). Figures 4(b,c) show m-mode views of the single-cycle videos reconstructed at 1x, 2x, 4x, and 8x temporal magnification using the NW kernel regression model with the proposed bivariate RBF kernel defined in both cardiac phase and frame similarity space vs a univariate RBF kernel defined in cardiac phase space only.

## III. Experimental Data and Results

We used 2D cardiac ultrasound or echocardiography videos and simultaneously captured ECG recordings of 6 anesthetized mice to validate our methods. The ultrasound videos were acquired using the VisualSonics Vevo 2100 scanner at 233 frames per second (FPS). Each video consists of approximately 300 frames, 11 cardiac cycles, and 2 respiratory cycles.

We validated the cardiac phase estimates of our method by comparing them with the cardiac phase derived from the ECG signal which is the gold standard for cardiac gating. Figure 1(a) presents a visual comparison between the cardiac phase derived from the ECG signal through linear interpolation between R-wave peaks [12], [13] and the cardiac phase estimated directly from the video using our method on one of the 6 videos. Figure 1(b) presents a visual comparison of five video frames evenly spaced in time between two consecutive R-wave peaks of the ECG signal (top-row) and the corresponding minima of the cardiac phase signal computed using our method (bottom-row). Table I reports statistics of the error between the cardiac phases estimated by our method and the cardiac phases derived from the ECG signal for all the 6 videos where in the phases are in the normalized [0,1] range.

Next, we compared the performance of our phase estimation method with three previously published methods, namely: Phase correlation approach of Sundar *et al*. [4], Manifold learning approach of Wachinger *et al*. [15], and the Masked PCA approach of Panayiotou *et al*. [16]. For the phase correlation approach, we applied a bandpass filter in the mice cardiac frequency range (310–840 bpm) to the phase correlation signal to extract the cardiac signal. For the manifold learning approach, we project the high-dimensional image sequence onto the second principal direction and apply a band-pass filter in the mice cardiac frequency range (310–840 bpm) to extract the cardiac signal. For the Masked PCA approach, we compute the binary mask by thresholding the response of Frangi’s vesselness filter tuned to enhance the moving heart chamber walls that look like ridges, perform PCA on the intensities of pixels within the binary mask, project the high-dimensional image sequence onto the second principal direction (projection onto the first principal direction only encodes respiratory motion in our data), and apply a band-pass filter in the mice cardiac frequency range to extract the cardiac signal. Note that each of these three prior works only propose a method for deriving a 1D signal reflecting the periodicity characteristics of the cardiac/respiratory motion in the given image sequence. They do not provide an approach to compute the instantaneous phase from the derived signal. Hence, we compare the performance of these methods with ours in localizing the R-wave peaks of the ECG signal that corresponds to the peaks/valleys of the 1D signals derived by these methods. Table II reports the *mean* ± *stddev* frame error of different methods in locating the video frames corresponding to the R-wave peaks of the ECG signal for all 6 videos where in the result of the best performing method for each video is highlighted in bold. Note that the average duration between two consecutive frames is 4.29 ms and the length of cardiac cycle in our videos is 27.27 frames or 116.98 ms.

Next, we validated the accuracy of our kernel regression model for reconstructing images at any cardiac phase using leave-one-out-cross-validation (LOOCV). In each round of cross-validation, we randomly pick one of the non-respiratory frames in the video, hold out the selected frame along with corresponding frames (closest in phase) in each cycle, fit our kernel-regression model on the remaining frames, use the fitted model to reconstruct the image at the cardiac phase of the held out frames, and compute the similarity between the reconstructed and original image using normalized correlation. The second column of Table III shows the *mean* ± *stddev* of normalized correlation between the reconstructed and the original images over 50 rounds of LOCCV for all 6 videos. As a *baseline*, the third column of Table III reports the *mean*±*stddev* of the normalized correlation between frames at R-wave peaks of the ECG signal.

**Fig. 5.**
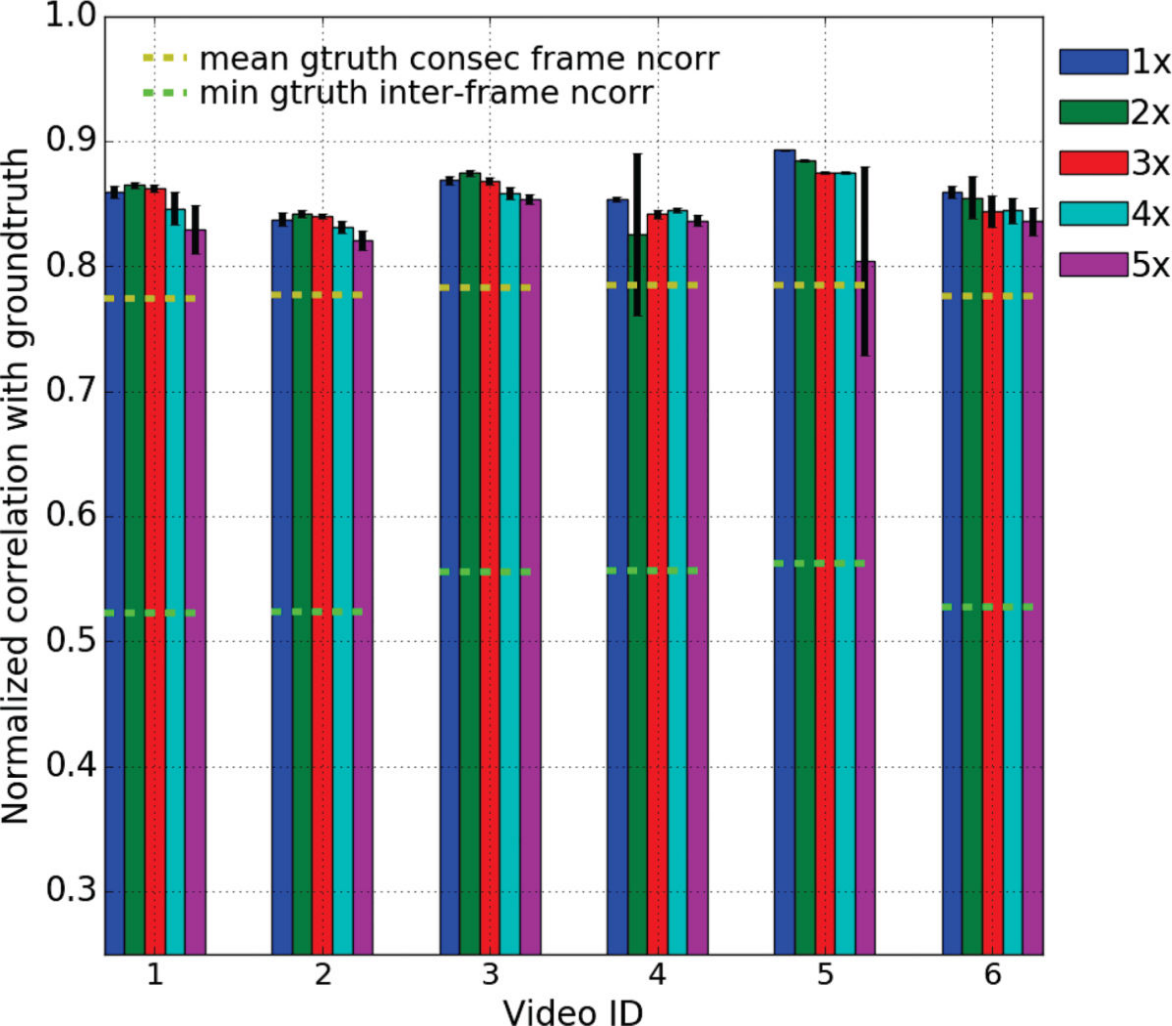
Evaluation of the quality of single-cycle videos reconstructed using our kernel regression model at increasing levels of temporal magnification: We down-sampled each of the 6 videos in our dataset by a series of factors between 1x-5x, reconstructed the single-cycle video back at the original resolution using our kernel regression model, and computed its similarity using normalized correlation (ncorr) with the ground truth created by cropping out the portion between two consecutive ECG R-wave peaks wherein respiratory motion is minimal. To avoid any bias to the starting phase, we created the down-sampled videos starting at 7 random points in the first 14 frames and computed the mean and stddev of the ncorr between the reconstructed and ground truth single cycle video. As a baseline, we show the mean ncorr among all consecutive frames (yellow dashed lines) and the min ncorr among all pairs of frames (green dashed lines) in the ground truth video.

Lastly, we evaluated the quality of the single cycle videos reconstructed using our kernel regression model at different levels of temporal magnifications as described below. For each of the 6 videos in our dataset, we created the groundtruth single cycle video by cropping out the portion between two consecutive ECG R-wave peaks wherein the respiratory motion is minimal. We then temporally down-sampled each video by increasing factors between 1x (233 FPS) and 5x (47 FPS), reconstructed the single cycle video back at the original temporal resolution using our kernel regression model, and computed its similarity with the ground truth using normalized correlation. To avoid any potential bias towards the phase of the starting frame, we created the down-sampling videos starting from 7 random points in the first 14 frames (equal to half of the cardiac cycle length) and computed the mean and standard-deviation (stddev) of the similarity of the reconstructed videos with the ground truth. Figure 5 shows the results of this experiments. Note that the frame rate of our videos is 233 FPS and the average heart rate of the mice in our dataset is 512 bpm. A 5x factor of down-sampling results in a 46 FPS video with only 5 frames per cardiac cycle beyond which the phase estimation begins to break down.

The supplementary material includes videos showing the instantaneous cardio-respiratory phases obtained using our phase-estimation method and the single-cycle videos at 1x, 2x, 4x, and 8x temporal magnification generated using our kernel regression model for one of the 6 videos in our dataset.

## IV. Conclusion

In this paper, we presented a novel method for retrospective estimation of instantaneous cardio-respiratory phases directly from cardiac ultrasound videos, thereby eliminating the need of additional hardware to track them in, for example, small animal studies. We also presented a robust non-parametric regression technique for gating out respiratory frames and a novel kernel regression model for reconstructing images at any cardiac phase to facilitate temporal super-resolution.

We next plan to evaluate our methods on more datasets and address remaining pitfalls. Our phase estimation method makes a strong assumption of periodicity which may not hold in the case of subjects with cardiac arrhythmia. To address this, we will look into univariate methods for phase estimation in quasi-periodic signals that are more resilient to noise [18], [19], [26]. The use of normalized correlation to measure interframe similarity relies on an inherent assumption that a large part of the image is pulsating. To relax this, we will explore local patch-based similarity measures. Lastly, the local kernel weighted average in our NW kernel regression model may cause blurring at high temporal magnifications. We plan to alleviate this using a manifold kernel regression approach wherein the weighted average is computed in a diffeomorphic registration sense [27]. Source code^1^ of the proposed methods and the data^2^ used to validate them are available online.

## Supplementary Material

tbme-chittajallu-2823279-mm

## Figures and Tables

**TABLE I:** Error between the cardiac phases estimated using our method and those obtained from the ECG signal.

VID	mean ± stddev	median	IQR	range
1	0.06 ± 0.03	0.06	0.05	[0.00, 0.14]
2	0.06 ± 0.03	0.05	0.05	[0.00, 0.12]
3	0.06 ± 0.03	0.06	0.06	[0.00, 0.12]
4	0.04 ± 0.02	0.04	0.02	[0.00, 0.10]
5	0.06 ± 0.03	0.06	0.03	[0.00, 0.12]
6	0.03 ± 0.02	0.04	0.03	[0.00, 0.05]
Mean	0.05 ± 0.03	0.05	0.04	[0.00, 0.11]

**TABLE II:** Error in localization of the frames corresponding to the peaks of the R-wave in ECG signal using different methods.

	mean ± stddev error in frames			
VID	Proposed	Phase corr [14]	Manifold learn [15]	Mask PCA [16]
1	**1.09 ± 1.08**	4.09 ± 2.97	1.45 ± 0.78	1.55 ± 0.78
2	**1.36 ± 0.08**	2.91 ± 2.23	1.82 ± 0.94	1.73 ± 1.05
3	**1.18 ± 0.03**	3.45 ± 1.97	1.45 ± 1.08	1.55 ± 0.78
4	**0.73 ± 0.86**	4.64 ± 3.75	1.36 ± 0.98	1.18 ± 1.03
5	**1.27 ± 0.86**	4.70 ± 3.77	**1.27 ± 0.86**	1.36 ± 0.88
6	**0.80 ± 0.40**	6.00 ± 3.52	1.00 ± 0.63	1.10 ± 0.54
Mean	**1.07 ± 0.55**	4.30 ± 3.04	1.39 ± 0.87	1.41 ± 0.84

**TABLE III:** Evaluation of our kernel regression model for reconstructing images by cardiac phase using leave one out cross-validation (LOOCV) of 50 rounds.

VID	LOOCV (50) *mean* ± *stddev ncorr*	QRS Peak Frames *mean* ± *stddev ncorr*
1	0.82 ± 0.03	0.70 ± 0.06
2	0.81 ± 0.03	0.71 ± 0.05
3	0.81 ± 0.03	0.70 ± 0.04
4	0.84 ± 0.03	0.74 ± 0.06
5	0.85 ± 0.03	0.72 ± 0.06
6	0.85 ± 0.04	0.77 ± 0.07
Mean	0.83 ± 0.03	0.72 ± 0.05

## Footnotes

1 https://github.com/KitwareMedical/CardiacUltrasoundPhaseEstimation

2 https://data.kitware.com/#collection/599604148d777f7d33e9c43d
